# Plum supplementation and lipid profile: a systematic review and meta-analysis of randomised controlled trials

**DOI:** 10.1017/jns.2022.101

**Published:** 2023-01-16

**Authors:** Moein Askarpour, Hamid Ghalandari, Leila Setayesh, Ehsan Ghaedi

**Affiliations:** 1 Department of Clinical Nutrition, School of Nutritional Sciences and Dietetics, Shiraz University of Medical Sciences, Shiraz, Iran; 2 Department of Community Nutrition, School of Nutritional Sciences and Dietetics, Shiraz University of Medical Sciences, Shiraz, Iran; 3 Department of Nutrition and Health Sciences, University of Nebraska-Lincoln, Lincoln, NE; 4 Department of Clinical Nutrition, School of Nutritional Sciences and Dietetics, Tehran University of Medical Sciences, Tehran, Iran

**Keywords:** Lipid profile, Meta-analysis, Plum, Randomised controlled trials

## Abstract

Plums are abundant in bioactive compounds which have been associated with numerous health benefits. In the present study, we aimed at examining the impact of plum supplementation on lipid profile of individuals. Electronic bibliographical databases were searched for relevant randomised clinical trials. Articles meeting our eligibility criteria were included for data extraction and final analysis. Weighted mean difference (WMD) was estimated using a random-effect model. Of the total articles retrieved in the initial search, nine articles were found to be eligible to be included in the analysis. Our results show that plum supplementation significantly improves total cholesterols levels in the unhealthy individuals. Moreover, plum supplementation reduces the LDL-c levels in the pooled sample (WMD = −11⋅52 mg/dl; 95 % CI −21⋅93, −1⋅11, *P* = 0⋅03, *I*^2^ = 98⋅7 %) and also in some of the subgroups of individuals (dried plum, unhealthy subjects, duration more than 8 weeks). Moreover, it had a significant reducing effect on TC levels just in unhealthy subjects. Although plum supplementation did not have any significant impact on serum levels of TG nor HDL-c. Our results show that supplementation with plums is potentially effective in reducing serum total cholesterol and LDL-c.

## Introduction

The rate of nutrition-related non-communicable diseases is increasing worldwide^(1)^. Some possible explanations have been made to justify this phenomenon, including increase in the intake of ‘unhealthy’ foods and a reduction in physical activity^(2)^. Consequently, lifestyle alterations have led to an unprecedented rise in non-contagious, metabolic disorders; especially in the developed and developing countries. Lipid profile and its alterations have been used both as indicators of metabolic disorders and methods to evaluate patients’ response to dietary interventions^(3)^. There are some advantages to use lipid profile as screening, monitoring and evaluation biomarkers, including: (1) they are accessible at most healthcare facilities, (2) they are relatively inexpensive, hence more available to most patients and researchers, and (3) they show an acceptable level of sensitivity to lifestyle changes^(4–6)^. The impact of various lifestyle modifications on lipid profile of subjects has been investigated extensively; these interventions have included changes in physical activity, manipulations of macronutrient composition and the intake of individual foodstuffs^(7–9)^. Recently, researchers are more curious about the effect that different foodstuff may exert upon markers of metabolic disorders, including lipid profile^(10–13)^.

Plums are tree fruits belonging to the family of Rosaceae; a large family that consists of different species of other fruit, including apples, almonds, pears and so forth. These fruits are originally endemic to Asia, but currently are grown in different parts of the world^(14)^. Plums possess some characteristics that make them eligible to be examined regarding their possible health-inducing effects; including, but not limited to, their acceptable content of different micronutrients (thiamine, riboflavin, pyridoxine, ascorbic acid, retinol, γ-tocopherol, potassium, sodium, calcium, iron, copper, magnesium, phosphorus and selenium) and anti-oxidant agents (phytochemicals such as phenolic acids and flavonols)^(15,16)^. When looked as a whole, this fruit seems to be a good example of what experts in the field call a ‘functional food’^(17)^, for not only it consists of several significant essential nutrients, but it also provides extra benefits in the form of a potential potent anti-oxidant.

Due to these properties and their widespread consumption globally, researchers have investigated the possible impacts of plums on various markers of metabolic anomalies, including the lipid profile of patients. In this systematic review and meta-analysis, we aimed at finding the conducted studies in the form of randomised clinical trials (RCTs) and tried to sum them up to derive a clear answer as to the effectiveness of plums on improving parameters of lipid profile.

## Methods

The Preferred Reporting Items of Systematic Reviews and Meta-Analysis (PRISMA) statement guideline was applied to perform this meta-analysis^(18)^. Moreover, the parameters of PICOS-model^(19)^ including ‘population’ (adult subjects), ‘intervention’ (plum intake), ‘comparison’ (control group), ‘outcome’ (studies that reported total cholesterol (TC), low-density lipoprotein (LDL) cholesterol, high-density lipoprotein (HDL) cholesterol and triacylglycerols (TGs)) and ‘study design’ (randomised clinical trial) were determined.

### Search strategy

A comprehensive literature search for all published articles was done in electronic database including PubMed, Web of Science, Cochrane and Scopus from inception to November 2021. Various search terms such as MeSH and non-MESH terms used were as follows: (‘plum’, OR ‘Eugenia jambolana’, OR ‘Syzygium cumini’) AND (‘Intervention Studies’ OR ‘intervention’ OR ‘controlled trial’ OR ‘randomised’ OR ‘randomised’ OR ‘random’ OR ‘randomly’ OR ‘placebo’). The full search strategy is shown in Supplementary Table S1. To prevent missing any eligible studies, hand searches of all reference lists of relevant articles were also conducted.

### Eligibility criteria

The inclusion criteria in this systematic review and meta-analysis were as follows: (a) reported one of the following measures: TC, LDL, HDL, TG; (b) adult population; and (c) randomised control trials (RCTs). Articles were excluded if studies reported: (1) the impact of plum supplementation along with other interventions, (2) insufficient data for the outcomes of interest, (3) less than 2 weeks of intervention period and (4) subjects under 18 years old, pregnant and lactating women.

### Data extraction

The following data were obtained from included studies: author's name, publication's year, study location, study duration, study design, health status of study population, gender, number and mean age of participants, dose of plum intake, levels of lipid profile before and after intervention. Two independent researchers (M. A., E. Gh.) conducted the study selection (Table 1).

**Table 1.** Characteristics of included studies

Author (location, year)	Study design	Population	Gender	Intervention			Duration	Intervention/Control	Outcomes
				Number	Mean (range) age (years)	Dosage			
Ahmed *et al.* (Pakistan, 2010a)	RP	Pre-hypertensive adult patients	M/F	31	43	11⋅5 g/d	8 weeks	Dried plums in water/water	TC, LDL-c, HDL-c, TG
Ahmed *et al.* (Pakistan, 2010b)	RP	Pre-hypertensive adult patients	M/F	27	42	23 g/d	8 weeks	Dried plums in water/water	TC, LDL-c, HDL-c, TG
Bhaswanta *et al.* (Australia, 2019)	RP	Mildly hypertensive overweight or obese	M/F	16	47	250 ml/d	12 weeks	Queen Garnet (QG) plums/raspberry cordial	TC, LDL-c, HDL-c, TG
Chai *et al.* (USA, 2012)	RP	Healthy postmenopausal women	F	55	57⋅5	75 g/d	12 weeks	Dried plum/dried apple	TC, HDL-c, TG
Chiu *et al.* (Russia, 2017a)	RP	Mild hypercholesterolemic	M/F	20	18–53	50 ml/d	4 weeks	Prune essence concentrates (PEC)/simulated prune drink	TC, LDL-c, HDL-c, TG
Chiu *et al.* (Russia, 2017b)	RP	Mild hypercholesterolemic	M/F	20	18–53	100 ml/d	4 weeks	Prune essence concentrates (PEC)/simulated prune drink	TC, LDL-c, HDL-c, TG
Clayton *et al.* (USA, 2019)	RP	Healthy overweight adult	M/F	24	35⋅3	41⋅8 g per twice a day	8 weeks	Dried plum/low fat muffin	TC, LDL-c, HDL-c, TG
Howarth *et al.* (USA, 2010)	RC	Adult women	F	29	36⋅4	91⋅35 g/d	2 weeks	Dried plum/low fat muffin	TG
Santhakumar *et al.* (Australia, 2015)	RC	Healthy	M/F	13	30	200 ml/d	4 weeks	Queen Garnet plum juice/placebo juice	TC, LDL-c, HDL-c, TG
Tinker *et al.* (USA, 1991)	RC	Mild hypercholesterolemic	M/F	41	46⋅5	100 g/d	4 weeks	Prunes/grape juice	TC, LDL-c, HDL-c, TG
Tucakovica *et al.* (Australia, 2018)	RC	Healthy	M/F	20	41	200 ml/d	4 weeks	Queen Garnet plum juice (QGPJ)/placebo drink	TC, LDL-c, HDL-c, TG

M, male; F, female; RC, randomised crossover design; RP, randomised parallel design; HDL-c, high-density lipoprotein cholesterol; LDL-c, low-density lipoprotein cholesterol; TC, total cholesterol; TG, triacylglycerol.

### Quality assessment

Random allocation of participants was shown in all included trials. The method of random sequence generation has been described in four trials^(20–23)^, whereas the rest of the studies had an unclear risk of bias. Allocation concealment of six trials^(20–25)^ has been reported and other studies indicated unclear risk of bias. Risk of bias regarding blinding of participants and personnel and blinding of outcome assessment in three trials was judged as low^(20,22,23)^. All of the studies showed low risk of bias based on incomplete outcome data and selective reporting. Also, all of the trials had unclear risk of bias based on other potential threats to validity. Details of risk of bias assessment are shown in Table 2.

**Table 2.** Cochrane risk of bias of included studies

Author (location, year)	Sequence generation	Allocation concealment	Blinding of participants and personnel	Blinding of outcome assessment	Incomplete outcome data	Selective outcome reporting	Other potential threats to validity
Ahmed *et al.* (Pakistan, 2010)	U	U	U	U	L	L	U
Bhaswanta *et al.* (Australia, 2019)	L	L	L	L	L	L	U
Chai *et al.* (USA, 2012)	U	U	U	U	L	L	U
Chiu *et al.* (Russia, 2017)	U	U	U	U	L	L	U
Clayton *et al.* (USA, 2019)	U	L	U	U	L	L	U
Howarth *et al.* (USA, 2010)	L	L	U	U	L	L	U
Santhakumar *et al.* (Australia, 2015)	L	L	L	L	L	L	U
Tinker *et al.* (USA, 1991)	U	L	U	U	L	L	U
Tucakovica *et al.* (Australia, 2018)	L	L	L	L	L	L	U

L, low risk; H, high risk; U, unknown.

### Statistical analysis

The meta-analysis was conducted to compare the pooled estimates of lipid profile before and after the intake of plum supplementation. Mean and standard deviation (sd) of lipid profile parameters were used to describe the pooled effect; otherwise, standard errors (se) were changed to sd using the formula: [(se*√*n*); *n* is the study sample size]. The sensitivity analysis was performed using a leave-one-out method for included studies, to detect the impact of each study on the overall estimate of effect size^(26)^. Publication bias was detected using Begg's rank correlation test and Egger's weighted regression^(27,28)^. A random-effect model (Dersimonian-Liard), as well as subgroup analysis, was applied to pool the effect sizes, in case of high heterogeneity between studies. Statistical analyses were performed using Stata software version 14 (StataCorp. College Station, Texas, USA). *P*-value < 0⋅05 was considered as statistically significant.

## Results

### Study selection

**Fig. 1.**
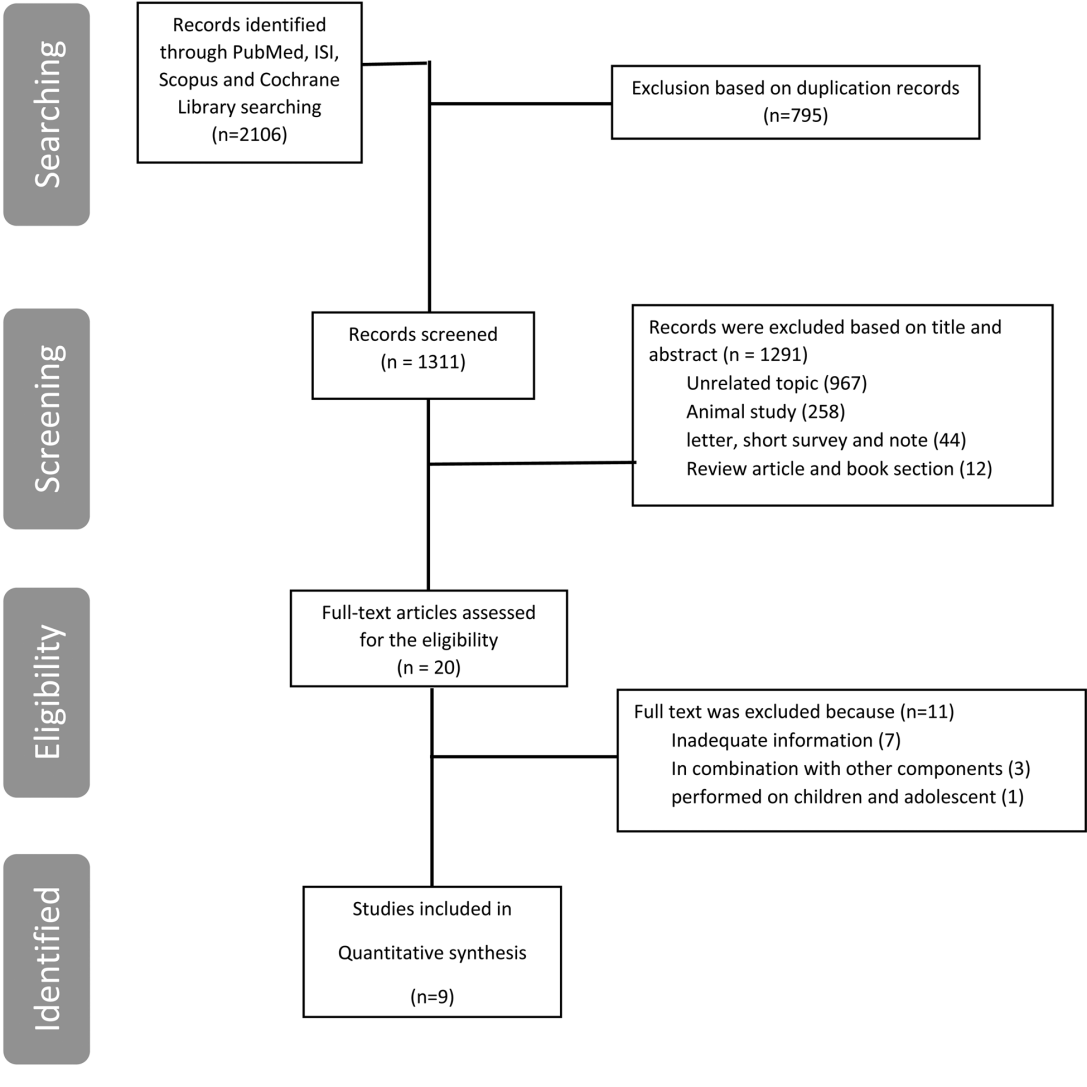
Flow diagram of study selection.

From the initial search from different databases, 2106 studies were retrieved (512 from PubMed, 611 from ISI, 195 from Cochrane and 788 from Scopus). After removing duplicates (*n* = 795), 1311 articles remained. Then, 1291 were excluded after careful assessment of the titles and abstracts based on PICOS criteria, for the following reasons: (1) unrelated title (*n* = 967), (2), animal studies (*n* = 258), (3) review articles and book sections (*n* = 12) and (4) other reasons (letter, short survey and note) (*n* = 44). Finally, twenty potentially relevant articles were obtained for full-text evaluation and detailed assessment. Eleven full-text articles were omitted, due to the following reasons: insufficient data on the outcomes of interest (*n* = 7), performed on children and adolescents (*n* = 1), combined intervention along with plum supplementation (*n* = 3). Finally, a total of nine eligible studies met the inclusion criteria. The PRISMA flow diagram is described in Fig. 1.

### Characteristics of the studies

The detailed characteristics of the nine included studies with eleven arms are described in Table 1. Overall, 592 participants were randomly assigned and completed the studies. Of the nine trials included in the present meta-analysis, two trials were conducted on women^(21,29)^ and seven trials^(20,22–25,30,31)^ on both sexes. The included studies varied in the range from 2 to 12 weeks. The study design of five trials was parallel^(20,24,29–31)^, while crossover designs were used in other studies^(21–23,25)^. Supplementation was carried out using plum's powder drink in six studies^(20,22,23,25,30,31)^ and dried plum in the rest^(21,24,29)^. In matching control groups, different interventions were used, including dried apple^(29)^, water^(30)^, raspberry cordial^(20)^, simulated prune drink^(31)^, low fat muffin^(21,24)^, placebo juice^(22,23)^ and grape juice^(25)^. Participants with various health statuses were enrolled in the clinical trials, such as hypertensive patients^(20,30)^, hypercholesterolemic patients^(25,31)^ and healthy subjects^(21–24,29)^. The included studies were conducted in a range of locations, including Pakistan^(30)^, Australia^(20,22,23)^, USA^(21,24,25,29)^ and Russia^(31)^.

### Effect of plum supplementation on TC

**Fig. 2.**
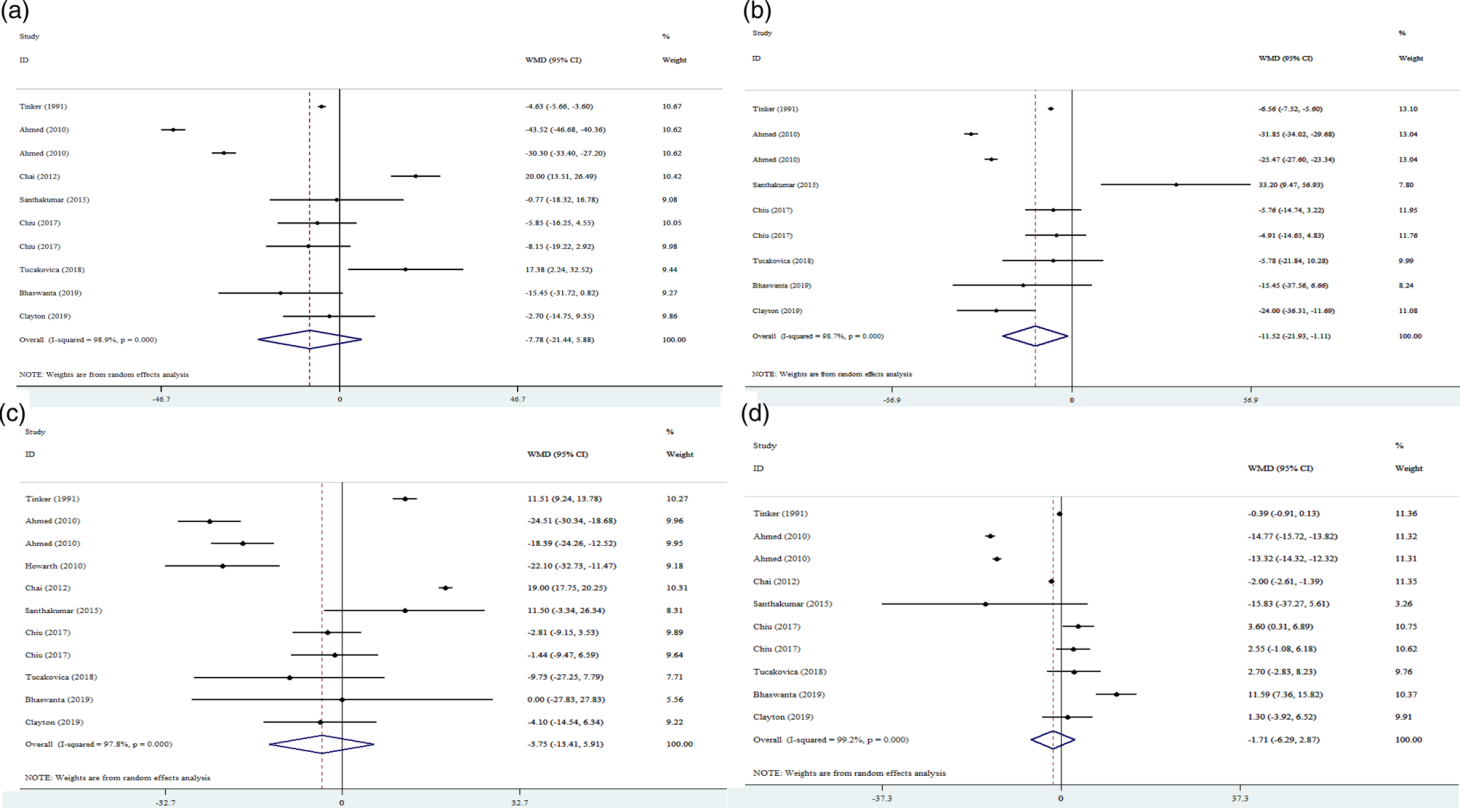
Forest plot for the effect of plum supplementation on TC (a), LDL-cholesterol (b), TG (c) and HDL-cholesterol (d) concentrations, expressed as mean differences between intervention and control groups. Horizontal lines represent 95 % CIs. Diamonds represent pooled estimates from random-effects analysis.

The final result of the meta-analysis with 10 arms of RCTs (362 cases and 400 control subjects) showed that plum supplementation had no significant effect with considerable heterogeneity between studies on TC serum levels (Fig. 2(a)). Subgroup analyses (Table 3) were applied to reduce the heterogeneity, thus the articles were categorised based on type of intervention (dried plum *v*. plum juice), age (≤42 *v*. >42), health status (healthy *v*. unhealthy), duration (<8 *v*. ≥8 weeks) and the number of participants (≤45 *v*. >45). Plum supplementation was observed to be effective in reducing TC levels only in unhealthy subjects.

**Table 3.** Subgroup analysis to assess the effect of plum intake on lipid indices

Group	No. of trials	WMD (95 % CI)	*P*-value^a^	*I*^2^ (%)^b^	*P*-heterogeneity^c^	*P* for between subgroup heterogeneity^d^
TC						
Intervention						<0⋅001
Dried plum	4	−0⋅28 (−11⋅02, 10⋅46)	0⋅959	62⋅6	0⋅046	
Plum Juice	6	−12⋅91 (−31⋅04, 5⋅22)	0⋅163	99⋅4	<0⋅001	
Health status						<0⋅001
Healthy	4	−18⋅24 (−35⋅90, −0⋅57)	0⋅043	99⋅3	<0⋅001	
Unhealthy	6	−12⋅91 (−31⋅04, 5⋅22)	0⋅157	77⋅9	0⋅004	
Duration (week)						<0⋅001
≤8 weeks	5	−2⋅45 (−8⋅74, 3⋅83)	0⋅445	54⋅2	0⋅068	
>8 weeks	5	−14⋅62 (−35⋅71, 6⋅47)	0⋅175	98⋅8	<0⋅001	
Mean age						<0⋅001
≤42	4	−4⋅74 (−29⋅03, 19⋅53)	0⋅343	95⋅1	<0⋅001	
>42	6	−9⋅60 (−29⋅44, 10⋅23)	0⋅702	99⋅2	<0⋅001	
Number of the study						<0⋅001
≤45	6	−3⋅73 (−9⋅70, 2⋅23)	0⋅220	52⋅1	0⋅064	
>45	4	−14⋅42 (−38⋅13, 9⋅28)	0⋅223	99⋅1	<0⋅001	
LDL						
Intervention						<0⋅001
Dried plum	4	−21⋅88 (−36⋅85, −6⋅90)	0⋅004	99⋅5	<0⋅001	
Plum juice	5	−1⋅99 (−12⋅47, 8⋅50)	0⋅710	62⋅6	0⋅03	
Health status						<0⋅001
Healthy	3	−0⋅45 (−29⋅46, 28⋅56)	0⋅976	88⋅9	<0⋅001	
Unhealthy	6	−15⋅29 (−27⋅63, −2⋅94)	0⋅015	99⋅2	<0⋅001	
Duration (week)						<0⋅001
≤8 weeks	5	−3⋅26 (−10⋅05, 3⋅53)	0⋅346	63⋅3	0⋅028	
>8 weeks	4	−27⋅32 (−32⋅78, −21⋅86)	<0⋅001	84	<0⋅001	
Mean age						<0⋅001
≤42	4	−8⋅74 (−26⋅80, 9⋅31)	0⋅343	89⋅5	<0⋅001	
>42	5	−12⋅96 (−28⋅49, 2⋅56)	0⋅102	99⋅1	<0⋅001	
Number of the study						<0⋅001
≤45	6	−4⋅23 (−10⋅53, 2⋅06)	0⋅188	56⋅6	0⋅042	
>45	3	−27⋅99 (−33⋅59, −22⋅40)	<0⋅001	88⋅5	<0⋅001	
TG						
Intervention						<0⋅001
Dried plum	6	−8⋅55 (−30⋅52, 13⋅41)	0⋅584	86⋅2	<0⋅001	
Plum juice	5	2⋅52 (−6⋅43, 11⋅46)	0⋅445	98⋅8	<0⋅001	
Health status						<0⋅001
Healthy	5	−0⋅78 (−19⋅46, 17⋅91)	0⋅935	95⋅3	<0⋅001	
Unhealthy	6	−6⋅28 (−21⋅14, 8⋅57)	0⋅407	97⋅4	<0⋅001	
Duration (week)						<0⋅001
≤8 weeks	6	−1⋅79 (−12⋅44, 8⋅87)	0⋅742	91⋅8	<0⋅001	
>8 weeks	5	−5⋅79 (−29⋅81, 18⋅22)	0⋅636	98⋅9	<0⋅001	
Mean age						<0⋅001
≤42	5	0⋅59 (−10⋅55, 11⋅74)	0⋅917	98⋅2	<0⋅001	
>42	6	−9⋅54 (−20⋅21, 1⋅13)	0⋅080	97⋅8	<0⋅001	
Number of the study						<0⋅001
≤45	7	−1⋅64 (−11⋅73, 8⋅46)	0⋅608	90⋅2	<0⋅001	
>45	4	−6⋅94 (−33⋅46, 19⋅58)	0⋅750	99⋅1	<0⋅001	
HDL						
Intervention						<0⋅001
Dried plum	5	−6⋅02 (−12⋅10, 0⋅07)	0⋅053	99⋅6	<0⋅001	
Plum juice	5	4⋅33 (−0⋅09, 8⋅76)	0⋅55	75⋅0	0⋅003	
Health status						<0⋅001
Healthy	6	−1⋅96 (−9⋅30, 5⋅38)	0⋅747	99⋅5	<0⋅001	
Unhealthy	4	−0⋅54 (−3⋅85, 2⋅76)	0⋅601	48⋅5	0⋅120	
Duration (week)						<0⋅001
≤8 weeks	5	1⋅44 (−1⋅18, 4⋅06)	0⋅281	63⋅5	0⋅027	
>8 weeks	5	−3⋅72 (−10⋅97, 3⋅52)	0⋅314	99⋅5	<0⋅001	
Mean age						<0⋅001
≤42	4	−5⋅10 (−16⋅15, 5⋅95)	0⋅366	94⋅9	<0⋅001	
>42	6	−0⋅17 (−5⋅37, 5⋅04)	0⋅950	99⋅3	<0⋅001	
Number of the study						<0⋅001
≤45	6	3⋅21 (−0⋅85, 7⋅26)	0⋅121	87⋅7	<0⋅001	
>45	4	−7⋅41 (−6⋅29, 2⋅87)	0⋅061	99⋅6	<0⋅001	

WMD, weighted mean difference; CI, confidence interval.

a Refers to the mean (95 % CI).

b Inconsistency, percentage of variation across studies due to heterogeneity.

c Obtained from the *Q*-test.

d Obtained from the fixed-effects model.

### Effect of plum supplementation on LDL-c

To assess the impact of plum supplementation on serum LDL-c levels, nine arms of RCTs (307 cases and 355 control subjects) were analysed. Plum supplementation was found to significantly reduce serum levels of LDL-c, using an inverse variance method. Studies also had substantial heterogeneity (Fig. 2(b)). The same subgroup analysis (Table 3) was applied for this variable; the results showed that LDL-c levels dropped significantly in the following subgroups including dried plum, unhealthy subjects and studies with more than 8 weeks follow-up.

### Effect of plum supplementation on triacylglycerol

Using pooled effect size on 11 arms of trial studies (391 cases and 429 control subjects), the results illustrated that plum supplementation exerts no significant effect on TG levels. Moreover, a significant between-study heterogeneity was seen (Fig. 2(c)). All of the aforementioned subgroup analyses (Table 3) were also conducted to address the possible source of heterogeneity. The same lack of beneficial effects was observed following these analyses.

### Effect of plum supplementation on HDL-c

Ten arms of clinical randomised trials (362 cases and 400 control subjects) were included to investigate the changes of serum HDL-c levels following plum supplementation. No meaningful effect on the levels of HDL-c was detected using pooled effect size, with a significant between-study heterogeneity (Fig. 2(d)). Subgroup analysis (Table 3) confirmed that plum supplementation does not lead to any significant improvements in serum HDL-c concentrations.

### Sensitivity analysis

We conducted the sensitivity analysis for all of the parameters of our interest (TG, TC, LDL-c and HDL-c), and the results showed that the elimination of none of studies could alter the effect size.

### Publication bias

**Fig. 3.**
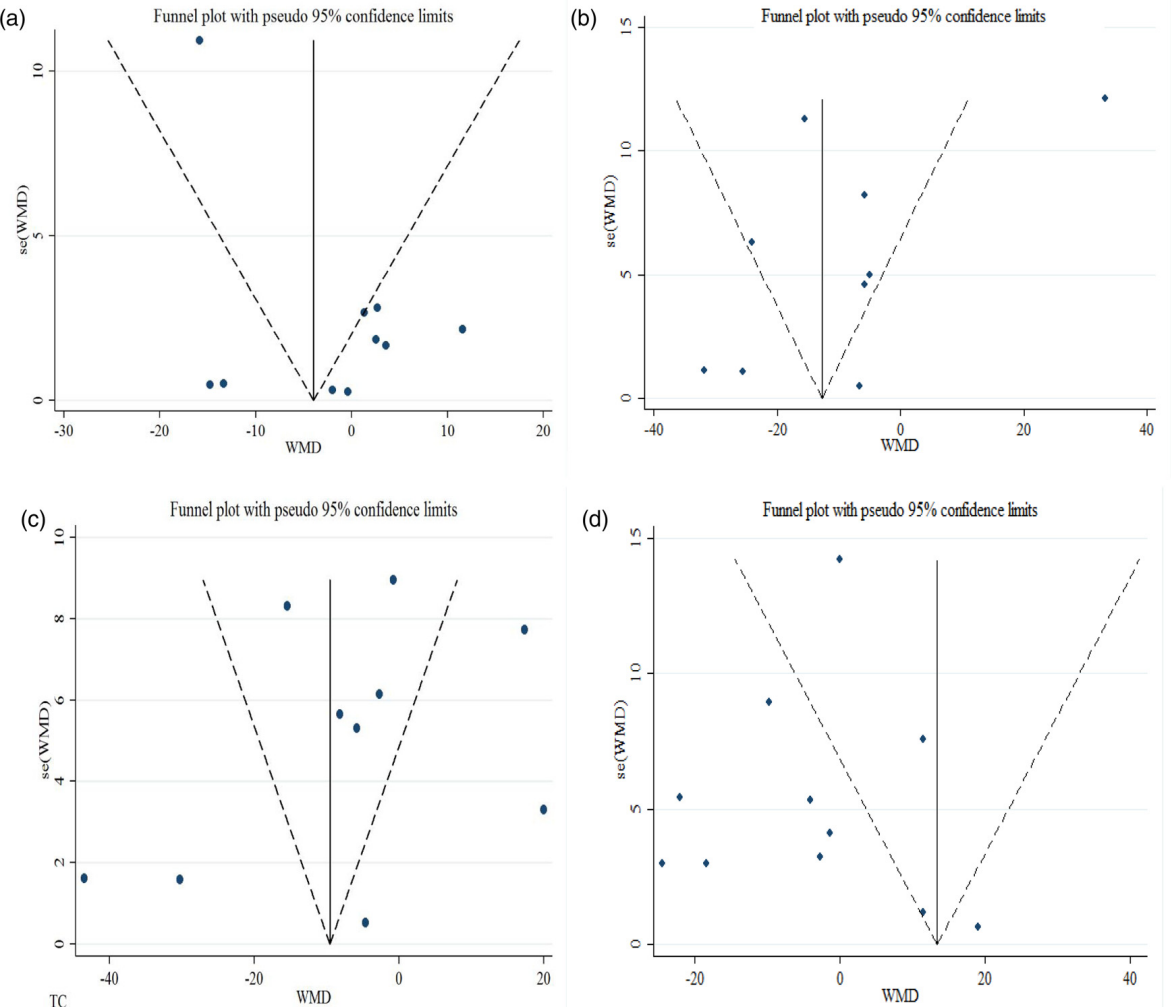
Funnel plots for publication bias: (a) HDL-cholesterol, (b) LDL-cholesterol, (c) TC and (d) TG.

All of the included studies were assessed for publication bias by Begg's rank correlation and Egger's weighted regression tests. Visual inspection of funnel plots (Fig. 3) showed slight asymmetry. However, the outcomes of Begg's test did not show any publication bias for TC (*P* = 0⋅898), TG (*P* = 0⋅436), LDL-c (*P* = 0⋅754) and HDL-c (*P* = 0⋅283). The results of Egger's test also indicated no publication bias for HDL-c (*P* = 0⋅937), LDL-c (*P* = 0⋅624) and TC (*P* = 0⋅685). Based on Egger's regression test, we found publication bias for TG (*P* = 0⋅009), so we did trim and filled analysis for TG and found that the overall effect size did not change (WMD = −3⋅75 mg/dl; 95 % CI −13⋅41, 5⋅91).

### Grading of evidence

The GRADE framework was used to show the quality of evidence for outcomes. The quality of evidence, based on the GRADE protocol, was ranked into four levels: high, moderate, low and very low^(32)^. Based on the GRADE protocol, the evidence about the outcomes was determined as moderate quality for LDL, low quality for HDL and TC and very low quality for TG (Supplementary Table S2).

## Discussion

In this systematic review and meta-analysis, we verified the possible impact of plum supplementation on lipid profile in nine eligible RCTs. Our findings suggest that plum can be beneficial in reducing TC in the unhealthy and LDL-c in all individuals. We were unable to detect any significant impact with regard to blood TG and HDL-c levels, both in crude and subgroup analyses.

As mentioned before, plums are rich sources of multiple phenolic compounds. Various flavonoids (anthocyanin, proanthocyanidin, procyanidin, catechin, quercetin and rutin), tannins and phenolic acids (hydroxycinnamic acids (HCAs), including chlorogenic, neochlorogenic, coumaric and caffeic acids) are among the most abundant bioactive phytochemicals in the plum fruit^(33)^. Each of these constituents have a unique and potent way of improving the health status of the individuals; as regards this study, their lipid profile.

Catechins, a group of chemicals belonging to the flavonoid family, has been shown to alter lipid and glucose metabolism by manipulating the adenosine monophosphate kinase (AMPK) pathway^(34)^ which, in turn, increases cellular energy metabolism. Anthocyanin, another flavonoid, has been shown to improve blood lipids by reducing serum TG, LDL-c levels and apolipoprotein B (apo-B), while increasing HDL-c and apolipoprotein A (apo-A) levels^(35,36)^. Experimental and cellular studies have proposed several mechanisms to explain these effects; including anthocyanins ability (1) to down-regulate hydroxymethylglutaryl coenzyme A (HMG-CoA) reductase, (2) to inhibit cholesteryl ester transporter (CETP), (3) to reduce the production/release of apo-B and apolipoprotein CIII (apo-CIII) and finally (4) to enhance the expression of LDL-c receptors^(35,37)^.

Previous studies have reported that HCAs, the other interesting compound with regard to plums’ activity in improving blood lipid profile, exert similar beneficial effects (reductions in TC and LDL-c)^(38)^. Augmented expression of peroxisome proliferator activator receptor-α (PPAR-α), enhancement of insulin activity, inhibition of HMG-CoA and acyl-CoA:cholesterol acyl transferase (ACAT) activity, and increased beta-oxidation and lipolytic activities are among the suggested mechanisms to elucidate this impact^(39–43)^. Lipid-lowering effects of HCAs have also been compared with that of pravastatin, a powerful cholesterol-lowering agent^(44)^.

In addition to their remarkable bioactive content, plums provide a good source of fibre in the diet. Firstly, fibres reduce the digestion and absorbability of important constituents of the diet, including lipids and carbohydrates by forming less soluble complexes and by physically sequestering them^(45,46)^; interestingly, this potential is doubled down by the same activity of some polyphenol compounds^(47,48)^. Furthermore, fibre content of plums and their other components having potential prebiotic features can modify gut environment in a way that would eventually lead to a reduction in lipogenesis in the body^(49,50)^. Previous cellular investigations have proposed that these features may be explained by the direct impact of SCFAs (produced by fermentation of fibres in the gut) on HMG-CoA reductase and the up-regulation of LDL-c receptors^(51)^. It must be noted that, besides fibres, polyphenolic compounds present in plums can, directly and independently, derive similar impacts^(52–54)^; thus, a possible synergistic empowerment could justify our observations in the study.

Moreover, multiple cellular and/or experimental studies have suggested that the manipulation of bile acids (BAs) metabolism is another possible way to explain the beneficial effects of plums observed in this review. Briefly, they have proposed these pathways: (1) augmentation of the expression of cholesterol 7-α hydroxylase, more commonly known as cytochrome P450 7A1 (CYP 7A1) – the enzyme active in BAs production from cholesterol – through farnesoid X receptor (FXR), nuclear factor-kappa B (NFκB) and extracellular-signal-regulated kinase (ERK) signalling pathways; (2) reduced production of BAs transporters in the gut lumen, (3) hindering the enterohepatic circulation of BAs by physical sequestration and (4) increasing the number of bacteria in the normal flora of the gut which have been shown to play a role in de-conjugation and hydrolysis of BAs^(51,55)^.

It goes without saying that multiple factors exist, other than the supplementation with a mere foodstuff, than could contort the normal blood lipid composition. For instance, the percentage of carbohydrate in the regimen and its type are significant determinants affecting one's lipid profile, especially blood TG and HDL-c levels^(56)^. In a systematic review and meta-analysis, Kim *et al.*^(57)^ explored the association between multiple foodstuff, macro- and micronutrients, dietary patterns, and dietary indices and cardiovascular disease (CVDs), blood pressure and lipid profile. They reported that, rather expectedly, lower consumption of carbohydrates and higher low-carbohydrate-diet scores were associated with reduced risk of elevated TG and lowered HDL-c levels. Contrarily, they observed that the intake of fruits (important source of dietary carbohydrates) reduced the risk of elevated TG levels. Curiously, they also found that the intake of other foodstuff, such as dairy/milk, sugar-sweetened beverages and even coffee could remarkably alter the risk of a disturbed lipid profile. This major meta-analysis of sixty-two observational studies was mentioned here to signify the major role other dietary factors might play as significant covariates in lipid profile modification that may/may not have been addressed and adjusted for in the included studies of the present study.

The present study had some limitations. We were unable to observe any significant impact of plum supplementation on TG and HDL-c. There might exist some possible explanation for this observation. We know that some important lifestyle variables might be considered when conducting an intervention to correct these indices of lipid profile; energy intake, intake of carbohydrates (both the absolute amount and its contribution in energy provision relative to other macronutrients) and physical activity are namely of great significance^(58,59)^. To study the pure impact of plum supplementation on TG and HDL-c, it is crucial that these variables remain constant both before and during the intervention. We hypothesise that the intervention applied (in this case, its pooled effect in the included studies) was strong enough to compel its impact on TC and LDL-c, but not strong enough to override the effect of these confounders on TG and HDL-c which are more easily and profoundly affected by them.

Moreover, the noticeable heterogeneity between the included studies makes it difficult to make any solid conclusion regarding the impact of plum on lipid profile. It is also worthy of note that the bioavailability of the bioactive compounds which are the main contributors to plum's possible health benefits, can vary greatly by factors of storage and processing^(60–63)^. One of the main sources of heterogeneity in the included studies might be the use of different products with different levels of available polyphenols. Moreover, the microbial composition of the host could influence the amount of absorbable polyphenols from various plum products^(64)^. As another limitation of the study, it should be noted that we did not register the protocol of study on the Prospero. To the best of the knowledge of the authors of this article, this is the first systematic review and meta-analysis trying to sum up the possible impact of plum supplementation of items of lipid profile.

Regarding the discrepancy between the observations made in this analysis and the existing evidence, we propose that further clinical trials using a standard protocol considering the product, its method of administration and its storage, be conducted in homogeneous populations to derive a solid, compelling statement as to their effectiveness in improving blood lipid profile of individuals.

## Conclusion

In this systematic review and meta-analysis, we observed that supplementation with plum can lower TC and LDL-c of individuals. We were not able to show such an impact on TG and HDL-c in overall and subgroup analysis. We suggest that further clinical trials, taking advantage of a standard protocol of plum supplementation, be conducted to elucidate the possible effect that plum supplementation might exert on lipid profile parameters.

## Supplementary material

For supplementary material accompanying this paper visit https://doi.org/10.1017/jns.2022.101. click here to view supplementary material
