# Supplementary material for: Plum supplementation and lipid profile: a systematic review and meta-analysis of randomised controlled trials
Source: J Nutr Sci. 2023 Jan 16;12:e6. doi: 10.1017/jns.2022.101 (PMC9869099; doi:10.1017/jns.2022.101)
Supplement: Supplementary file 1 [file S204867902200101Xsup001.docx]

**Supplementary Table 1**: Medical subject headings (MeSH) and non-MeSH keywords used to search relevant publications

| Concept 1 | "plum"[tiab] OR "Eugenia jambolana"[ MeSH ] OR “ Syzygium cumini”"[ MeSH ] OR ”Eugenia jambolana”[ MeSH ]OR “Plum Tree, Java"[ MeSH ] OR “Eugenia cumini”[ MeSH ] |
| --- | --- |
| Concept 2 | Intervention [tiab] OR Intervention Study [tiab] OR Intervention Studies [tiab] OR controlled trial [tiab] OR randomised [tiab] OR random [tiab] OR randomly [tiab]) OR placebo [tiab] OR assignment [tiab] OR randomized controlled trial [tiab] OR randomized clinical trial [tiab] OR randomized clinical trial [tiab] OR rct [tiab] OR blinded [tiab] OR double blind [tiab]) OR double blinded [tiab]) OR open-label [tiab]) OR trial [tiab] OR clinical trial [tiab] OR clinical trial [tiab] OR "Non-Randomized Controlled Trials"[tiab] OR "Non-Randomized Trials"[tiab]) OR "Non-Randomized "[tiab] OR "Clinical Trials as Topic"[Mesh] |

Without limitation to language or date of publication

The combination of keywords as mentioned above was used to search online databases. ("concept 1" AND "concept 2")

**Supplementary Table 2.** GRADE profile of plum supplementation on lipid profile

| **Outcomes** | **Risk of bias** | **Inconsistency** | **Indirectness** | **Imprecision** | **Publication Bias** | **Quality of evidence** |
| --- | --- | --- | --- | --- | --- | --- |
| **TG** | No serious limitations | Serious Limitations | No Serious Limitations | Serious limitations | Serious limitations | ⊕◯◯ ◯ Very Low |
| **HDL** | No serious limitations | Serious Limitations | No Serious Limitations | Serious limitations | No serious limitations | ⊕⊕◯ ◯ Low |
| **LDL** | No serious limitations | Serious Limitations | No Serious Limitations | No serious limitations | No serious limitations | ⊕⊕◯ ◯ Moderate |
| **TC** | No serious limitations | Serious Limitations | No Serious Limitations | Serious limitations | No serious limitations | ⊕⊕◯ ◯ Low |
